# Impact of Serum Uric Acid Lowering and Contemporary Uric Acid-Lowering Therapies on Cardiovascular Outcomes: A Systematic Review and Meta-Analysis

**DOI:** 10.3389/fcvm.2021.641062

**Published:** 2021-03-23

**Authors:** Hangying Ying, Hongdi Yuan, Xiaomei Tang, Wenpu Guo, Ruhong Jiang, Chenyang Jiang

**Affiliations:** ^1^Key Laboratory of Cardiovascular Intervention and Regenerative Medicine of Zhejiang Province, Department of Cardiology, Sir Run Run Shaw Hospital, Zhejiang University School of Medicine, Hangzhou, China; ^2^Department of Nursing, Sir Run Run Shaw Hospital, Zhejiang University School of Medicine, Hangzhou, China

**Keywords:** uric acid-lowering therapy, xanthine oxidase inhibitor, febuxostat, cardiovascular outcome, cardiovascular safety

## Abstract

**Objective:** This study aimed to evaluate the potential association between uric acid (UA) lowering and cardiovascular risk reduction among UA-lowering therapies in adults.

**Methods:** A systematic search for randomized controlled trials (RCTs) was conducted according to the protocol pre-registered in PROSPERO (No. CRD42020199259). We search for RCTs in PubMed, Embase, Web of Science, the Cochrane Library, and ClinicalTrials.gov up to July 1, 2020. A meta-analysis was performed using a fixed- or random-effects model.

**Results:** In total, 30 studies involving 18,585 hyperuricaemic patients were included. Xanthine oxidase inhibitor (XOI) therapy produced a 6.0% reduction in relative risk (RR) for major adverse cardiovascular events (MACEs). The use of febuxostat was associated with a higher risk of cardiovascular events (CVEs) (RR: 1.09, 95% CI 0.998–1.19, *I*^2^ = 0.0%), but the difference was not statistically significant. Allopurinol treatment was associated with a lower CVE risk (RR: 0.61, 95% CI 0.46–0.80, *I*^2^ = 21.0%). Among the UA-lowering therapies, the drug treatments were associated with all-cause mortality (RR: 1.20, 95% CI 1.02–1.41, *I*^2^ = 0.0%). The subgroup with a UA endpoint <7 mg/dl was not associated with a higher CVE risk (RR: 0.57, 95% CI 0.35–0.92, *I*^2^ = 0.0%), and in the subgroup with a UA endpoint <5 mg/dl group, a lower risk of CVEs was not observed (RR: 0.99, 95% CI 0.69–1.44, *I*^2^ = 0.0%).

**Conclusions:** UA reduction caused by XOIs reduced the incidence of MACEs. UA-lowering medicines were associated with changes in all-cause mortality but not cardiovascular outcomes. The lower UA endpoint was not associated with reduced cardiovascular risk.

## Introduction

UA is a widely accepted risk factor for cardiovascular disease (CVD) as demonstrated by epidemiological and Mendelian randomization studies (1, 2). The First National Health and Nutrition Examination Survey (NHANES I) study demonstrated that an elevated UA level was associated with coronary heart disease-related mortality (3). Additionally, several studies found that lowering the UA level could decrease CVD morbidity and mortality (4, 5). UA has been well-described as an end-product of purine catabolism that contributes to the development of hypertension, chronic kidney disease, and CVD. Similarly, the presence of UA could act as an oxygen radical scavenger that protects the cardiovascular system against excessive oxidative damage (6). Several cohort studies also showed higher cardiovascular mortality in both low and high UA strata groups, assuming U- or J-shaped curves (6).

Hyperuricemia is defined as serum UA levels >7.0 mg/dl in men and >5.7 mg/dl in women; the therapeutic target for gout is <6.0 mg/dl (7). The latest UA-lowering drugs, which including uricosuric medications, xanthine oxidase inhibitors (XOIs) and uricases, offer more alternatives for controlling UA levels (8). However, UA-lowering medicines exert unpredictable effects, including increasing the cardiovascular risk, leading to rare but fatal adverse events. In 2019, due to the results regarding cardiovascular safety from the CARES study, the United States Food and Drug Administration (FDA) issued a black box warning on febuxostat, which is used as a first-line treatment for hyperuricaemia. The available clinical evidence suggests that CVEs were more common in the intervention group, in which lesinurad plus febuxostat were administrated, than in the control group, in which placebo plus febuxostat were administrated (9).

Therefore, we performed this study to further explore the relationship between UA-lowering therapies and cardiovascular risk. The main purpose of this meta-analysis was to evaluate the association between UA lowering and cardiovascular risk reduction using different UA-lowering agents and to determine whether a cut-off value for the greatest cardiovascular benefit exists. We also discussed the effects of different UA-lowering agents on cardiovascular outcomes and mortality in patients with hyperuricaemia.

## Methods

### Data Sources and Searches

This study was conducted according to a pre-specified protocol registered at the International Prospective Register of Systemic Reviews (CRD42020199259). Relevant randomized controlled trials (RCTs) in databases, including PubMed, Embase, Web of Science, the Cochrane Library, and ClinicalTrials.gov, published from inception to July 1, 2020, were considered. We read the references of the included studies to identify additional relevant articles. The primary search terms we used included the following: (hyperuricemia) or (uric acid-lowering) and (xanthine oxidase inhibitors or uricosurics or uricases or purine nucleotide phosphorylase inhibitors) and (cardiovascular outcomes or major adverse cardiovascular event or mortality) and (randomized controlled trial). The specific search strategies are shown in Supplementary Table 2.

### Study Selection

We used the following prespecified inclusion and exclusion criteria for the trials. We included trials if they were (1) RCTs that included more than 50 patients who received UA-lowering medicines, with at least a 4-weeks follow-up period; (2) phase II-IV clinical trials in which the participants were adults (age>18 years); and (3) clinical trials that reported the serum UA levels and clinical outcomes. The clinical outcomes included CVD morbidity and mortality, hospitalization, and all-cause mortality. We excluded trials in which (1) the main pharmacological effects of the drugs were not UA lowering and the drugs had certain effects on cardiovascular outcomes (i.e., angiotensin receptor antagonists, losartan, calcium channel blockers, fenofibrate, and sodium-dependent glucose transporter 2 inhibitors); (2) the enrolled participants did not suffer life-threatening diseases (i.e., tumor or end-stage renal disease); and (3) the studies involved benzbromarone, due to serious adverse effects, including liver dysfunction.

### Data Extraction and Quality Assessment

As shown in Supplementary Table 1, two reviewers independently used a standard extraction template to extract the following items, including the following: (1) general information of the study (trial name/first author, year of publication, population characteristics, number of patients, age, sex proportion, and follow-up period); and (2) specific information of the study (drug information in the intervention group, drug information in the control group, baseline UA and the reduction in UA in each group, UA level achieved in each group and the difference between the groups). The difference in the achieved UA level between the groups was calculated as the mean or median difference. We also extracted mortality or hospitalization results if reported.

In our study, the cardiovascular outcomes were divided into two categories, including major adverse cardiovascular events (MACEs) and cardiovascular events (CVEs). We defined MACEs as cardiovascular death, non-fatal myocardial infarction, and non-fatal stroke (10). The number of MACEs which including cardiovascular death, non-fatal myocardial infarction, and non-fatal stroke in each study was extracted if it was reported. To achieve a better understanding of CVDs, we also defined CVEs according to the Anti-Platelet Trialists' Collaboration (APTC) (11) as cardiovascular death, non-fatal myocardial infarction, non-fatal stroke, angina, coronary revascularization, cerebral revascularization, congestive heart failure, arrhythmia (no evidence of ischaemia), venous and peripheral arterial vascular thrombotic events, transient ischaemic attack, and other non-APTC CVEs.

We defined intervention group as a more potent pharmacological UA-lowering strategy, whereas control group which regard as a less intensive UA- lowering therapy corresponded to placebo/usual care or the active control group of the trial. Among febuxostat studies, we defined allopurinol treatments or placebo as control groups. Among allopurinol studies, treatments with placebo were considered as control groups. Among more potent pharmacological agents like lesinurad, verinurad, oxypurinol, and pegloticase, treatments with febuxostat or allopurinol were used as control groups.

Two independent reviewers used the Cochrane Risk-of-Bias Assessment Tool to assess the potential risk of bias in all RCTs. If disagreement occurred, arbitration by another person was sought.

### Data Synthesis and Analysis

The data were analyzed according to the intention-to-treat principle, and the results are presented as a fitted line in a meta-regression and as an RR with a 95% confidence interval (CI). Regarding the cardiovascular and clinical outcomes, we performed a meta-analysis using a fixed-effects or random-effects Mantel-Haenszel model according to the level of heterogeneity. Meta-regression analyses were used to evaluate the association between UA reduction and cardiovascular risk. These analyses were conducted using random-effects models with the restricted maximum likelihood estimation. The Knapp and Hartung adjustments were applied to estimate the standard errors of the estimated coefficients to calculate the summary effect estimates. Regrading to cut-off values for the cardiovascular benefits, we conducted subgroup analyses by categorizing the endpoint level of serum UA. Sensitivity analyses were also performed, including a meta-analysis performed by the “leave-one-out” method, analyses of studies with subjects with cardiovascular comorbidities, analyses of high-quality studies with sample sizes ≥200 and follow-up periods ≥24 weeks, and analyses of hyperuricaemic subjects with and without symptoms.

In our study, the between-group heterogeneity was quantified using the Cochrane Q statistics, with the values of 25, 50, and 75% representing low, moderate, and high degrees of heterogeneity, respectively. We used a random-effects model if there was considerable level of heterogeneity (*I*^2^ more than 50%); otherwise, a fixed-effect model was used. Publication bias was assessed using funnel plots, and Egger's weighted regression was used to test for asymmetry. The statistical analysis was performed by Metafor package version 2.40 (R Project for Statistical Computing) and RevMan version 5.3 (Cochrane Collaboration).

## Results

**Figure 1:**
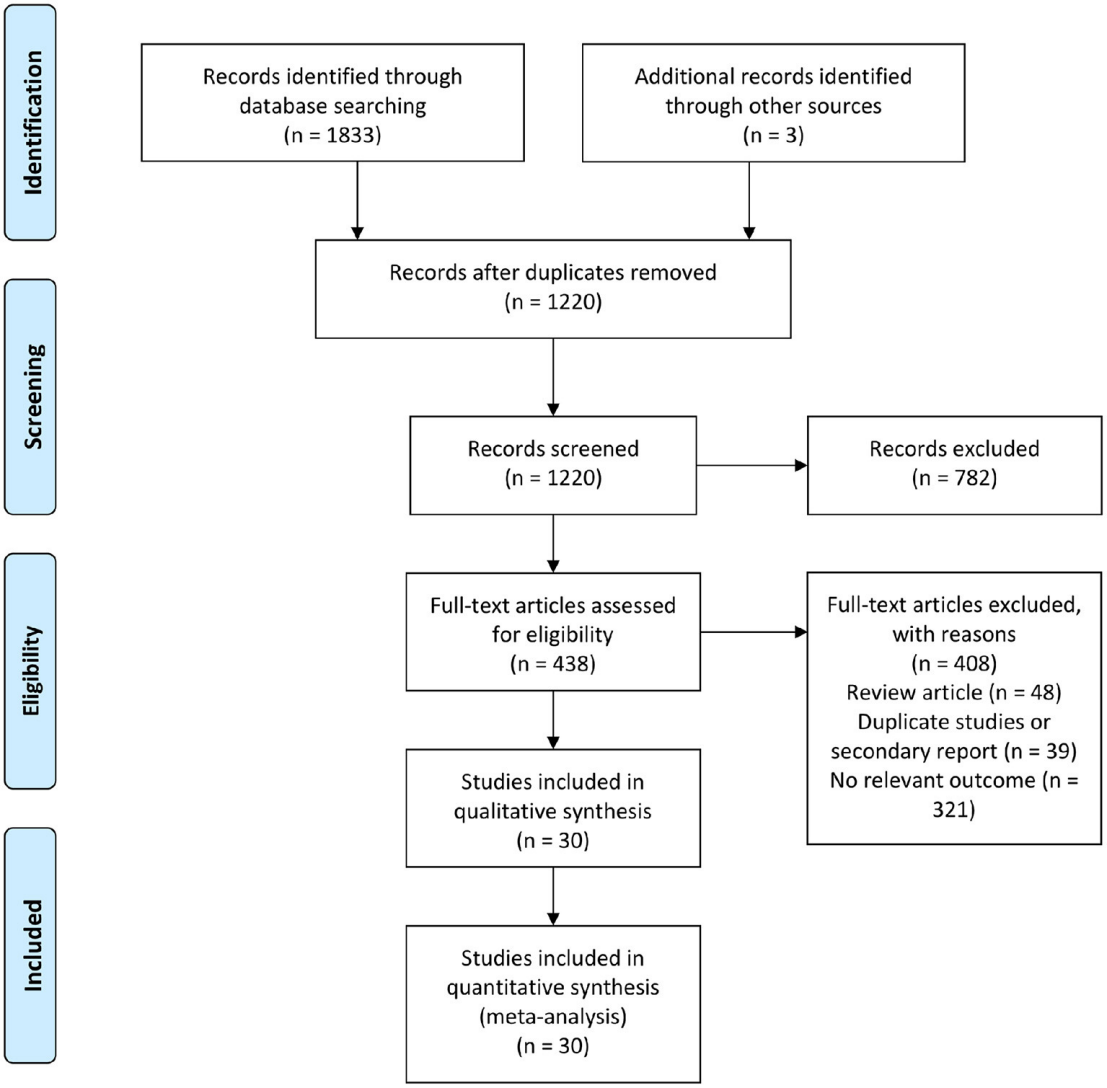
Flow diagram of the evidence search and selection process.

In total, 1,836 citations were retrieved by the initial database search, and 438 of these citations were identified and reviewed for eligibility. After reviewing the full-text, 30 studies were ultimately included in the qualitative synthesis and meta-analysis (Figure 1). In total, there were 14 studies involved febuxostat (14,572 patients), 8 studies involved allopurinol (1,270 patients), 4 studies involved lesinurad (1,751 patients), 2 studies involved verinurad (375 patients), 1 study involved oxypurinol (405 patients), and 1 study involved pegloticase (212 patients). The baseline characteristics of the included studies are presented in Supplementary Table 1.

### Cardiovascular Risk

**Figure 2:**
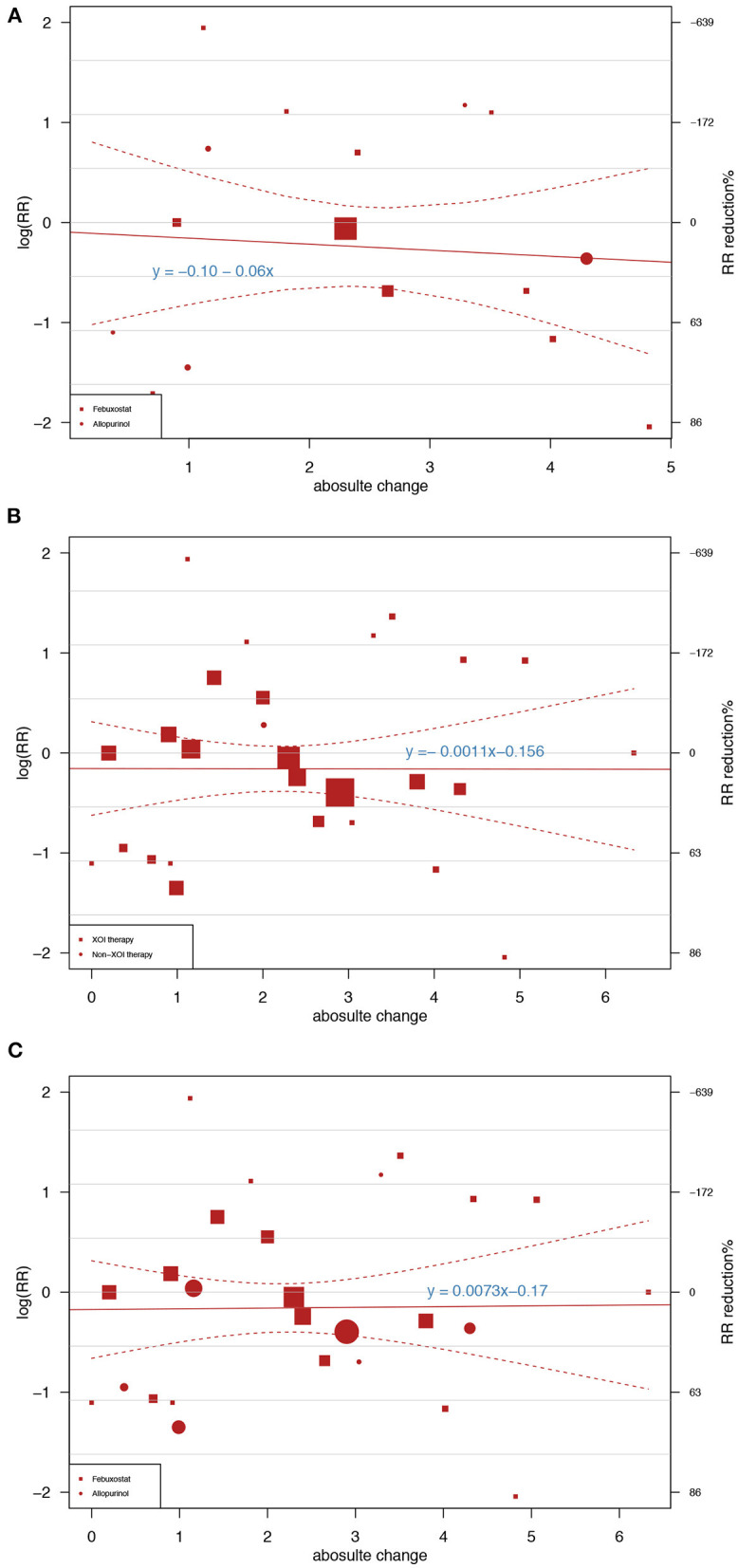
Meta-regression showing the association between the achieved uric acid (UA) differences between groups and the risk ratios of major adverse cardiovascular events (MACEs) and cardiovascular events (CVEs). The square and circle sizes are proportional to the study weight. The equation shown in the figure represents the meta-regression slope, which is drawn with a solid line, and the 95% confidence intervals, which are indicated by the dashed lines. **(A)** Association between UA reduction caused by xanthine oxidase inhibitors (XOIs) and MACEs. **(B)** Association between UA reduction caused by different UA-lowering medicines and CVEs. **(C)** Association between UA reduction caused by XOIs and CVEs.

In total, 12 trials involving febuxostat (8,261 patients), 7 trials involving allopurinol (1,217 patients), and 2 studies involving verinurad (375 patients) simultaneously reported the serum UA level and cardiovascular outcomes. Regarding MACEs as cardiovascular outcomes, of the 19 XOIs included in the trials, each 1 mg/dl (59.52 μmol/l) reduction in the UA level was associated with an average 6.0% CV risk reduction (y = −0.06x −0.10, Figure 2A). When treating the CVEs as cardiovascular outcomes, the results of the UA-lowering therapies and XOIs were similar, and the linear prediction was almost horizontal in the UA reduction range, as shown in both Figures 2B,C. As shown in Figure 2B, the RR per 1-mmol/l reduction was associated with a 0.11% cardiovascular risk reduction in different UA-lowering therapies (y = −0.0011x −0.156, Figure 2B). As shown in Figure 2C, the subjects who received XOIs exhibited a 0.73% increase in the cardiovascular risk per 1 mg/dl reduction in the UA level (y = 0.0073x−0.17, Figure 2C).

### Cardiovascular Outcomes

**Figure 3:**
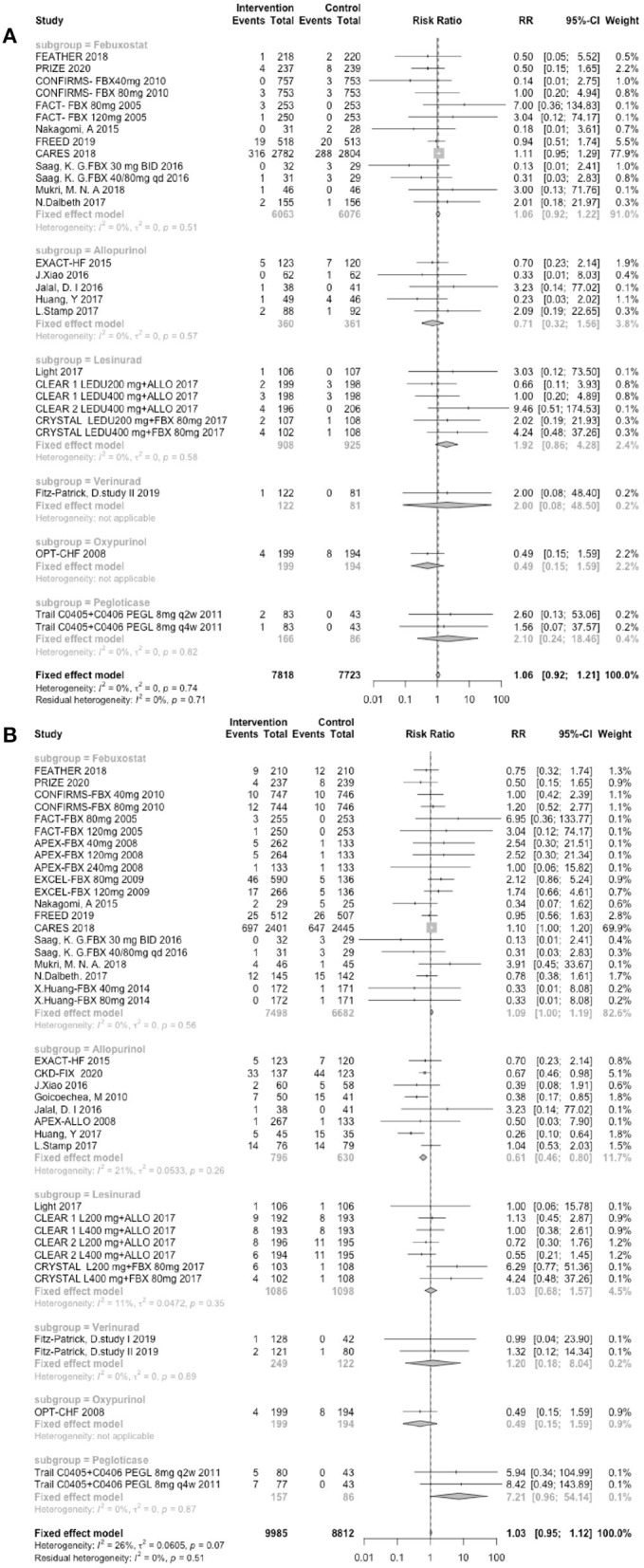
Assessment of cardiovascular outcomes. **(A)** Forest plots depicting the incidence of MACEs. **(B)** Forest plots depicting the incidence of CVEs. RR indicates risk ratio; CI indicates confidence interval.

In total, 13 trials involving febuxostat (14,451 patients), 7 trials involving allopurinol (1,217 patients), 4 studies involving lesinurad (1,751 patients), 2 studies involving verinurad (375 patients), 1 study involving oxypurinol (405 patients), and 1 study involving pegloticase (212 patients) that reported the cardiovascular outcomes. The results were divided into the following two categories: MACEs and CVEs. Compared with the control groups, the groups treated with different UA-lowering medicines were not associated with greater incidences of MACEs (RR: 1.06, 95% CI 0.92–1.21, *I*^2^ = 0.0%, *P* = 0.41, Figure 3A) or CVEs (RR: 1.03, 95% CI 0.95–1.12, *I*^2^ = 26.0%, *P* = 0.43, Figure 3B). As shown in Figure 3A, the subgroup analysis results were consistent with the main results. The different UA-lowering medicines did not demonstrate a significant difference in the incidence of MACEs. The febuxostat group showed a tendency of a higher risk of CVEs (RR: 1.09, 95% CI 0.998–1.19, *I*^2^ = 0.0%, *P* = 0.136, Figure 3B), but the difference was not statistically significant. Allopurinol treatment was associated with a lower CVE risk (RR: 0.61, 95% CI 0.46–0.80, *I*^2^ = 21.0%, *P* = 0.004, Figure 3B). The result of cardiovascular outcomes are shown in Table 1.

**Table 1:** Meta-analysis of treatment effect of uric acid -lowering therapies on cardiovascular outcome.

**Uric acid -lowering therapy**	**Sample size**	**Number of effect size synthesized**	**Summary effect size**	***P*-value**	**I-squared**
**MACEs**					
Febuxostat	11,782	13	1.06 (0.92, 1.22)	0.43	0.0%
Allopurinol	743	5	0.71 (0.32, 1.56)	0.39	0.0%
Lesinurad	1,547	6	1.92 (0.86, 4.28)	0.11	0.0%
Verinurad	204	1	2.00 (0.08, 48.50)	0.67	NA
Oxypurinol	405	1	0.49 (0.15, 1.59)	0.23	NA
Pegloticase	212	2	2.10 (0.24, 18.46)	0.51	0.0%
All effects	14,893	28	1.06 (0.92, 1.21)	0.41	0.0%
**CVEs**					
Febuxostat	14,167	20	1.09 (0.998, 1.19)	0.136	0.0%
Allopurinol	1,595	8	0.61 (0.46, 0.80)	0.004	21.0%
Lesinurad	1,751	7	1.03 (0.68, 1.57)	0.88	11.0%
Verinurad	375	2	1.20 (0.18, 8.04)	0.85	0.0%
Oxypurinol	405	1	0.49 (0.15, 1.59)	0.23	NA
Pegloticase	212	2	7.21 (0.96, 54.14)	0.055	0.0%
All effects	18,505	40	1.03 (0.95, 1.12)	0.43	26.0%

### Clinical Outcomes

**Figure 4:**
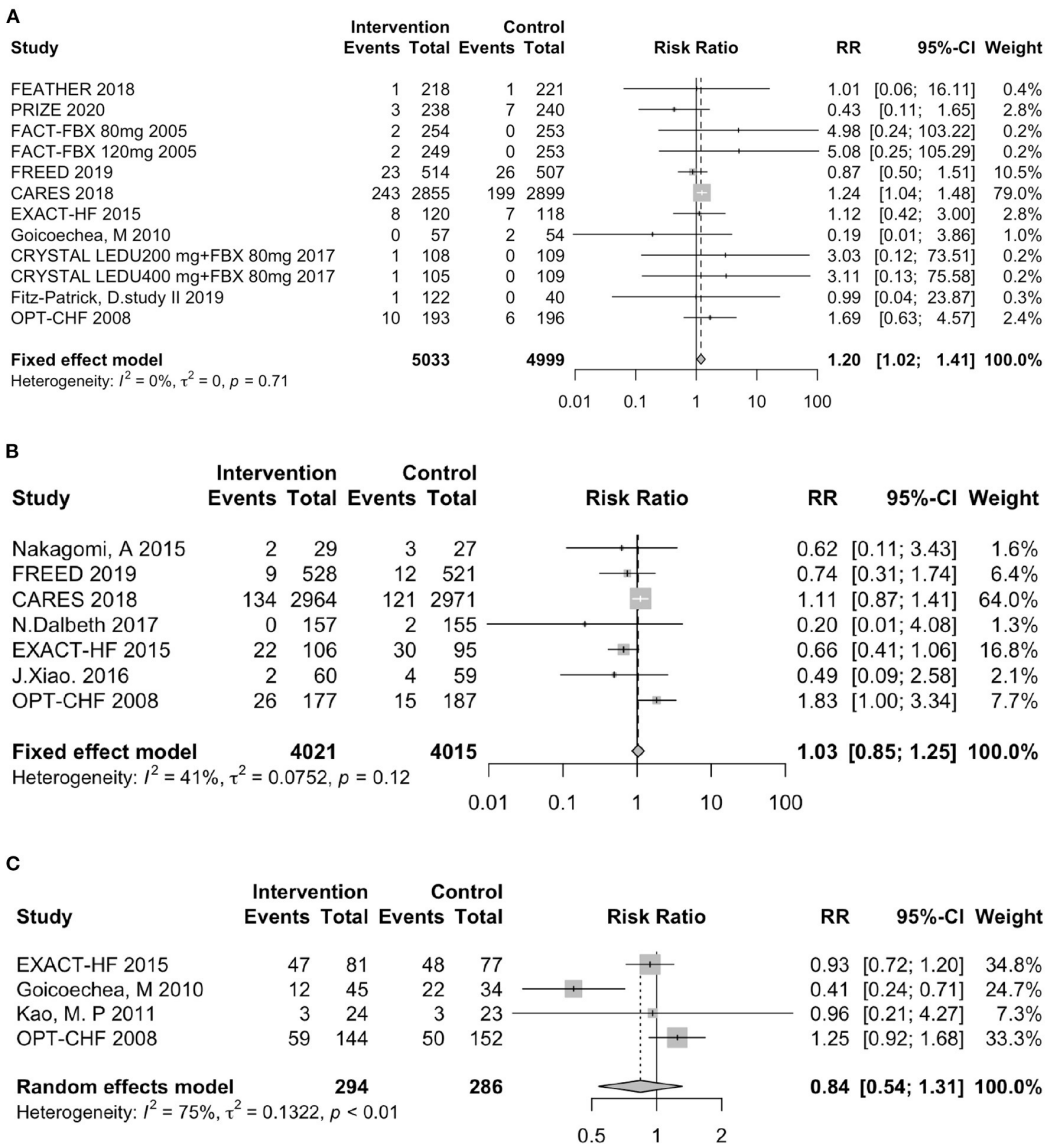
Assessment of clinical outcomes. **(A)** Forest plot of all-cause mortality. **(B)** Forest plot of heart failure-related hospitalization. **(C)** Forest plot of all-cause hospitalization. RR indicates risk ratio; CI indicates confidence interval.

In total, 7 trials involving febuxostat (9,319 patients), 4 trials involving allopurinol (544 patients), 1 study involving lesinurad (324 patients), 1 study involving verinurad (204 patients), and 1 study involving oxypurinol (405 patients) that reported the clinical outcomes. The clinical outcomes included the following three components: all-cause mortality (Figure 4A), heart failure hospitalization (Figure 4B), and all-cause hospitalization (Figure 4C). In the pooled data, the use of UA-lowering medicines was associated with a significant risk of all-cause mortality (RR: 1.20, 95% CI 1.02–1.41, *I*^2^ = 0.0%, *P* = 0.03). No difference was found between the groups treated with UA-lowering medicines and the control groups in terms of heart failure hospitalization (RR: 1.03, 95% CI 0.85–1.25, *I*^2^ = 41.0%, *P* = 0.74) or all-cause hospitalization (RR: 0.84, 95% CI 0.54–1.31, *I*^2^ = 75.0%, *P* = 0.44). The results of clinic outcomes are shown in Table 2.

**Table 2:** Meta-analysis of treatment effect of uric acid -lowering therapies on clinical outcome.

**clinical outcome**	**Sample size**	**Number of effect size synthesized**	**Summary effect size**	***P*-value**	**I-squared**
All-cause mortality	10,032	12	1.20 (1.02, 1.41)	0.03	0.0%
Heart failure hospitalization	8,036	7	1.03 (0.85, 1.25)	0.74	41.0%
All-cause hospitalization	580	4	0.84 (0.54, 1.31)	0.44	75.0%

### Uric Acid Endpoint

**Figure 5:**
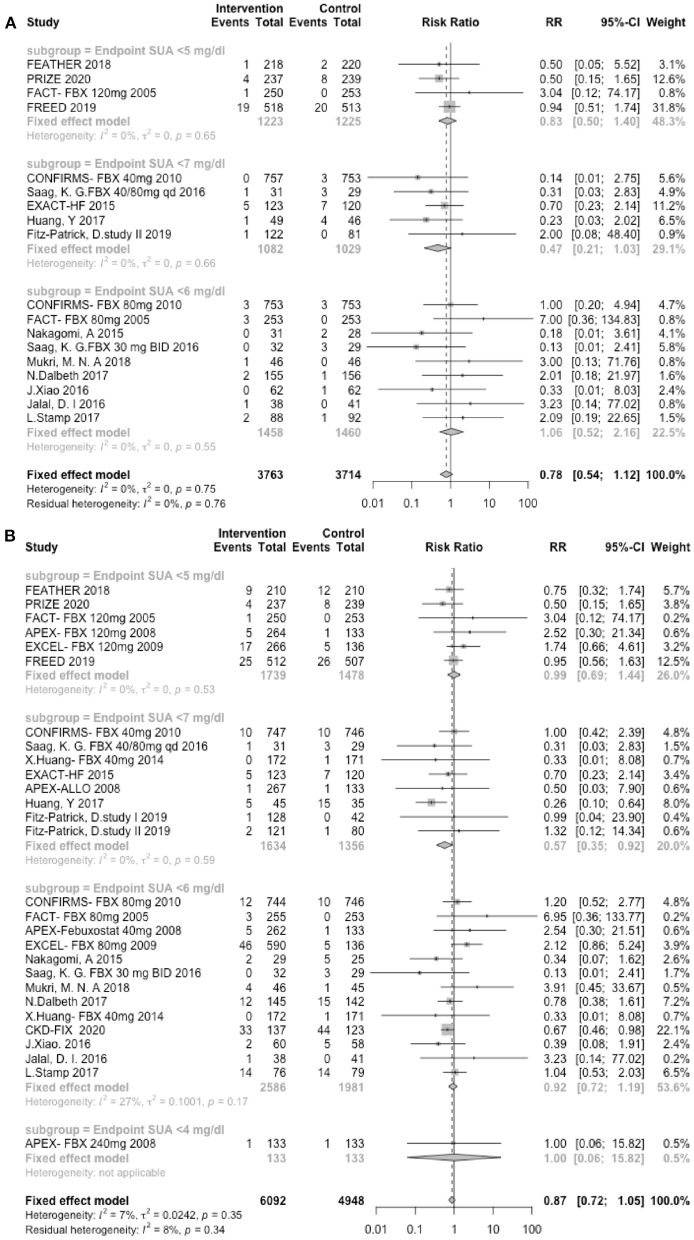
UA endpoints. **(A)** Forest plots depicting the incidence of MACEs according to the UA endpoint stratification. **(B)** Forest plots depicting the incidence of CVEs according to the UA endpoint stratification. RR indicates risk ratios; CI indicates confidence interval.

The analysis of the UA endpoint included 12 trials involving febuxostat (8,261 patients), 7 trials involving allopurinol (1,217 patients), and 2 studies involving verinurad (375 patients). In our study, the lower UA endpoint was not associated with a lower risk of either MACEs or CVEs. As shown in Figure 5A, in the subgroups with serum a UA endpoint <7 mg/dl, the intervention group did not demonstrate a higher risk of MACEs (RR: 0.47, 95% CI 0.21–1.03, *I*^2^ = 0.0%, *P* = 0.06), whereas in the subgroups with a serum UA endpoint <5 mg/dl, the intervention group did not show a lower risk of MACEs (RR: 0.83, 95% CI 0.50–1.40, *I*^2^ = 0.0%, *P* = 0.70). Similarly, as presented in Figure 5B, the subgroup with a serum UA endpoint <7 mg/dl was not associated with a higher risk of CVEs (RR: 0.57, 95% CI 0.35–0.92, *I*^2^ = 0.0%, *P* = 0.02), and in the subgroups with a serum UA endpoint <5 mg/dl, the intervention group did not show a lower risk of CVEs (RR: 0.99, 95% CI 0.69–1.44, *I*^2^ = 0.0%, *P* = 0.98).

### Sensitivity Analyses

We performed sensitivity analyses according to the “leave-one-out” method, analyses of studies with subjects with cardiovascular or cardiorenal comorbidities, and analyses of high-quality studies. As shown in Supplementary Figures 3A,B, as expected, the CARES study markedly contributed to the heterogeneity, while the other studies were highly consistent. Interestingly, the use of UA-lowering medicines decreased the cardiovascular risk in both patients with cardiovascular comorbidities (RR: 0.56, 95% CI 0.33–0.94, *I*^2^ = 65.0%, *P* = 0.028, Supplementary Figure 4) and those with cardiorenal comorbidities (RR: 0.65, 95% CI 0.44–0.94, *I*^2^ = 57.0%, *P* = 0.024, Supplementary Figure 5). In the analyses of high-quality studies, the results were consistent with the main findings (Supplementary Figure 6).

## Discussion

Since the first discovery of the association between UA and cardiovascular risk in 1967 in the Framingham cohort, the importance of UA in CVD has become increasingly evident. The increased serum UA level has a similar prognostic impact in CVE and all-cause mortality, whether caused by increased generation or reduced excretion (12). Several studies have suggested that UA serves as an independent risk factor for CVD, that UA-lowering medicines improved cardiovascular outcomes, and that lowering the UA level is one of the most effective methods for reducing cardiovascular risk (1, 13). However, both UA-lowering agents and very low levels of UA are threats to cardiovascular health. Due to the more frequent cardiovascular-related deaths in the CARES study, the FDA issued a black box warning on the use of febuxostat in 2019 (14). The very low UA level caused by newer UA-lowering agents might not competently inhibit the antioxidant effects of reactive oxygen species (ROS) (15). Further studies are needed to determine whether UA-lowering therapy is necessary in asymptomatic hyperuricaemia.

The present comprehensive study evaluated the association between UA and cardiovascular outcomes. The study was divided into three parts, and each part addressed one clinical conundrum. In the first part, we performed meta-regression analyses to explore the relationship between lowering UA and cardiovascular risk reduction. As shown in Figure 2, each 1 mg/dl reduction led to a 6.0% decrease in the MACE risk in the XOI-therapy group and a non-significant change in the CVE risk. In contrast to lipid-lowering therapy, excessive reduction was not associated with clinical benefits in our study. In another meta-analysis, each 1 mg/dl increase in UA resulted in a 5.35% increased risk of cardiovascular-mortality in type 2 diabetes patients (16). The number was 13.0% in the meta-analysis, which included 29 cohort studies involving 958,410 participants. The dose-response analysis demonstrated that the increase in the risk of mortality was associated with the female (RR = 2.44 [1.69–3.54]) but not male (RR = 1.02 [0.84–1.24]) sex (17). In our study, we used a narrow definition (MACE) and a broad definition (CVE) of cardiovascular outcomes. MACEs included cardiovascular death, non-fatal myocardial infarction, and non-fatal stroke, and these CV outcomes can all be improved by lowering the UA levels (15). Evidence suggesting that lower UA levels are associated with improved outcomes in some CVEs (i.e., arrhythmia, venous and peripheral arterial vascular thrombotic events, and transient ischaemic attack) is scarce. MACE symptoms are typical and rarely missed, but the non-specific symptoms of CVEs (i.e., transient ischaemic attack, thrombotic events, and arrhythmia) can be easily neglected. Therefore, the incidence of CVEs may have been underestimated, which is another reason why no significant difference was found between UA lowering and cardiovascular risk reduction in the CVE analysis.

In the second part, we conducted subgroup analyses to evaluate the cardiovascular risk and clinical outcomes of different UA-lowering medicines. XOIs, including allopurinol and febuxostat, are the first-line UA-lowering medications in clinical practice (13). Allopurinol is considered an inexpensive and effective drug for lowering the UA levels, and allopurinol treatment reduced cardiovascular events (RR: 71%) and hospitalizations (RR: 62%) compared with the control treatment (18). The use of febuxostat has recently been questioned as this agent is associated with an increased risk of all-cause and cardiovascular mortality (19). The subjects in the intervention group who received lesinurad plus febuxostat had a greater incidence of CVEs than those in the control group who received a placebo plus febuxostat (9). Similarly, the subjects in the groups receiving 8 mg of pegloticase both biweekly and monthly were at a higher risk of developing CVEs (20). In our study, the results demonstrated that CVEs were more common in the intervention groups receiving febuxostat than in the control group (RR: 1.09, 95% CI 0.998–1.19, *I*^2^ = 0.0%, *P* = 0.56), and the use of allopurinol decreased the CVE risk (RR: 0.61, 95% CI 0.46–0.80, *I*^2^ = 21.0%, *P* = 0.26). The patients receiving UA-lowering agents showed a higher risk of all-cause mortality (RR: 1.20, 95% CI 1.02–1.41, *I*^2^ = 0.0%, *P* = 0.71). However, these differences were not statistically significant. In the sensitivity analyses using by the “leave-one-out” method (Supplementary Figure 3), we found that the CARES study contributed to major heterogeneity in terms of the MACEs and CVEs. The FREED study, which was also designed to evaluate the effect of febuxostat in patients who are at a risk of cerebral and CVD, found limited evidence of cardiovascular risk with febuxostat use. Additionally, also shown in Supplementary Figure 3, the use of UA-lowering agents decreased cardiovascular risk in subjects with a history of CVD and cardio-renovascular diseases.

In the final part, to test the hypothesis that a lower UA level is associated with better cardiovascular outcomes and determine whether a cut-off value most influences the cardiovascular risk, we performed a subgroup analysis. As an illustration, the cardiovascular risks associated with different endpoints are shown in Figure 5. The results demonstrated that a lower UA level was not associated with better cardiovascular outcomes. Interestingly, the subjects in the group with a UA endpoint <7.0 mg/dl group, which is the upper limit of the normal value, exhibited a trend toward cardiovascular risk reduction. The subjects in the group with a UA endpoint <4.0 mg/dl group showed a trend toward poor outcomes. According to many cohort studies, the relationship between UA and all-cause or CVD mortality is U- or J- shaped (15). Regarding asian populations, a longitudinal cohort study conducted in China and Taiwan involving 127,771 adults reported that the CV-related mortality was higher for both UA levels <4 mg/dl (HR = 1.16 [1.07–1.25]) and ≥8 mg/dl (HR = 1.13 [1.06–1.21]) (21). In terms of European populations, the latest observational cohort study (URRAH Study), which enrolled 23,475 subjects with a follow-up period of at least 20 years, determined that the best cut-off value for cardiovascular mortality was 5.6 mg/dl, the optimal cut-off for fatal events was 5.70 mg/dl, and the favorable cut-off value for total mortality was 4.7 mg/dl (22, 23). The study also confirmed that there was a linear correlation between serum UA and an increased level of cardiovascular and all-cause mortality (23). As demonstrated in the subgroup analysis in Figure 3, the subjects in the CRYSTAL study with very low UA levels showed a greater incidence of CVEs (9). In contrast, those without very low UA levels did not have more CVEs in the other lesinurad studies (24–26). It has been reported that UA comprises up to 50% of the total antioxidant capacity of biological fluids in humans (27). UA serves as an oxygen radical scavenger that protects the cardiovascular system against excessive oxidative damage (6), which may be the explain for why very low UA levels are associated with poor cardiovascular outcomes.

Undoubtedly, we should provide UA-lowering agents to gout patients in clinical practice, but we should hesitate to provide these agents to asymptomatic hyperuricaemia patients. Consensus regarding the optimal treatment for asymptomatic hyperuricaemia is lacking. Many studies have shown strong associations between increased UA levels and poor CVE outcomes and cardiovascular-related death. In some epidemiological studies, a dose-response relationship was observed between elevated UA levels and poor outcomes, which appeared as a graded or linear relationship (28). Lowering the UA level is an effective method for improving outcomes, and regarding to cardiovascular and clinical outcomes, not all UA-lowering agents work equally (29); for example, allopurinol and febuxostat are also XOIs. In clinical practice, these agents must be used with caution.

### Limitations

There were several limitations that warrant acknowledgment. First, the trials included in this analysis are RCTs. Although RCTs provide high-quality evidence, and we combined all published data, the total number of people included was still small (18,585 patients). Second, the differences in the patients' backgrounds may lead to different cardiovascular outcomes. Some serious non-fatal diseases, including heart failure and chronic kidney diseases, may cause interference effects. Third, several definitions of CVEs were used in the included studies. In our studies, we defined MACEs as cardiovascular death, non-fatal myocardial infarction, and non-fatal stroke; the symptoms of the events are typical and rarely missed. However, according to our definition, CVEs include a larger range of events, and we extracted information according to study outcomes and treatment of emergent adverse events. Non-specific symptoms, including transient ischaemic attack, thrombotic events, and arrhythmia, can easily be neglected. Therefore, the incidence of CVEs may have been underestimated.

## Conclusion

In our study, the UA reduction caused by XOIs reduces the incidence of MACE but not the incidence of CVEs. The use of UA-lowering medicines was associated with a significant risk of all-cause mortality but not total RR for cardiovascular outcomes. The subgroup analyses provided evidence that febuxostat may be associated with a higher cardiovascular risk and that allopurinol treatments may decrease cardiovascular risk. Regarding the serum UA endpoints, a lower UA endpoint was not associated with reduced cardiovascular risk.

## Data Availability Statement

The original contributions presented in the study are included in the article/Supplementary Material, further inquiries can be directed to the corresponding author/s.

## Author Contributions

CJ and HYi were involved in the conception and design of the study. HYi and HYu collected the data. HYi, HYu, XT, WG, and RJ analyzed and interpreted the data and performed the meta-analysis. HYi, RJ, and CJ prepared the manuscript. All authors have approved the final manuscript.

## Conflict of Interest

The authors declare that the research was conducted in the absence of any commercial or financial relationships that could be construed as a potential conflict of interest.

## Supplementary Material

Supplementary Figure 1 Risk of bias graph.

Supplementary Figure 2 Funnel plot of the publication bias assessment **(A)** MACEs **(B)** CVEs.

Supplementary Figure 3 Sensitivity analysis of the “leave-one-out” method: **(A)** MACEs; **(B)** CVEs.

Supplementary Figure 4 Sensitivity analysis of the subjects with cardiovascular comorbidities.

Supplementary Figure 5 Sensitivity analysis of the subjects with cardiorenal comorbidities.

Supplementary Figure 6 Sensitivity analysis of the trials with a sample size ≥200 and follow-up period ≥24 w: **(A)** MACEs; **(B)** CVEs.

Supplementary Table 1 Baseline characteristics of the included studies.

Supplementary Table 2 Search strategy.
