# Corrigendum: Impact of Serum Uric Acid Lowering and Contemporary Uric Acid-Lowering Therapies on Cardiovascular Outcomes: A Systematic Review and Meta-Analysis

**DOI:** 10.3389/fcvm.2021.723626

**Published:** 2021-07-06

**Authors:** Hangying Ying, Hongdi Yuan, Xiaomei Tang, Wenpu Guo, Ruhong Jiang, Chenyang Jiang

**Affiliations:** ^1^Key Laboratory of Cardiovascular Intervention and Regenerative Medicine of Zhejiang, Department of Cardiology, Sir Run Run Shaw Hospital, Zhejiang University School of Medicine, Hangzhou, China; ^2^Department of Nursing, Sir Run Run Shaw Hospital, Zhejiang University School of Medicine, Hangzhou, China

**Keywords:** uric acid-lowering therapy, xanthine oxidase inhibitor, febuxostat, cardiovascular outcome, cardiovascular safety

There is an error in the Funding statement. The correct number for Ruhong Jiang is 81900289. The correct number for Chenyang Jiang is 81970269. The updated statement appears below.

“This study was supported by a grant from the National Natural Science Foundation of China (grant no. 81900289 and 81970269). The funders played no role in the study design, data collection or analysis, manuscript preparation, or decision to publish.”

The authors apologize for this error and state that this does not change the scientific conclusions of the article in any way. The original article has been updated.

