# Supplementary figures and images for: Impact of Serum Uric Acid Lowering and Contemporary Uric Acid-Lowering Therapies on Cardiovascular Outcomes: A Systematic Review and Meta-Analysis
Source: Front Cardiovasc Med. 2021 Mar 23;8:641062. doi: 10.3389/fcvm.2021.641062 (PMC8044896; doi:10.3389/fcvm.2021.641062)

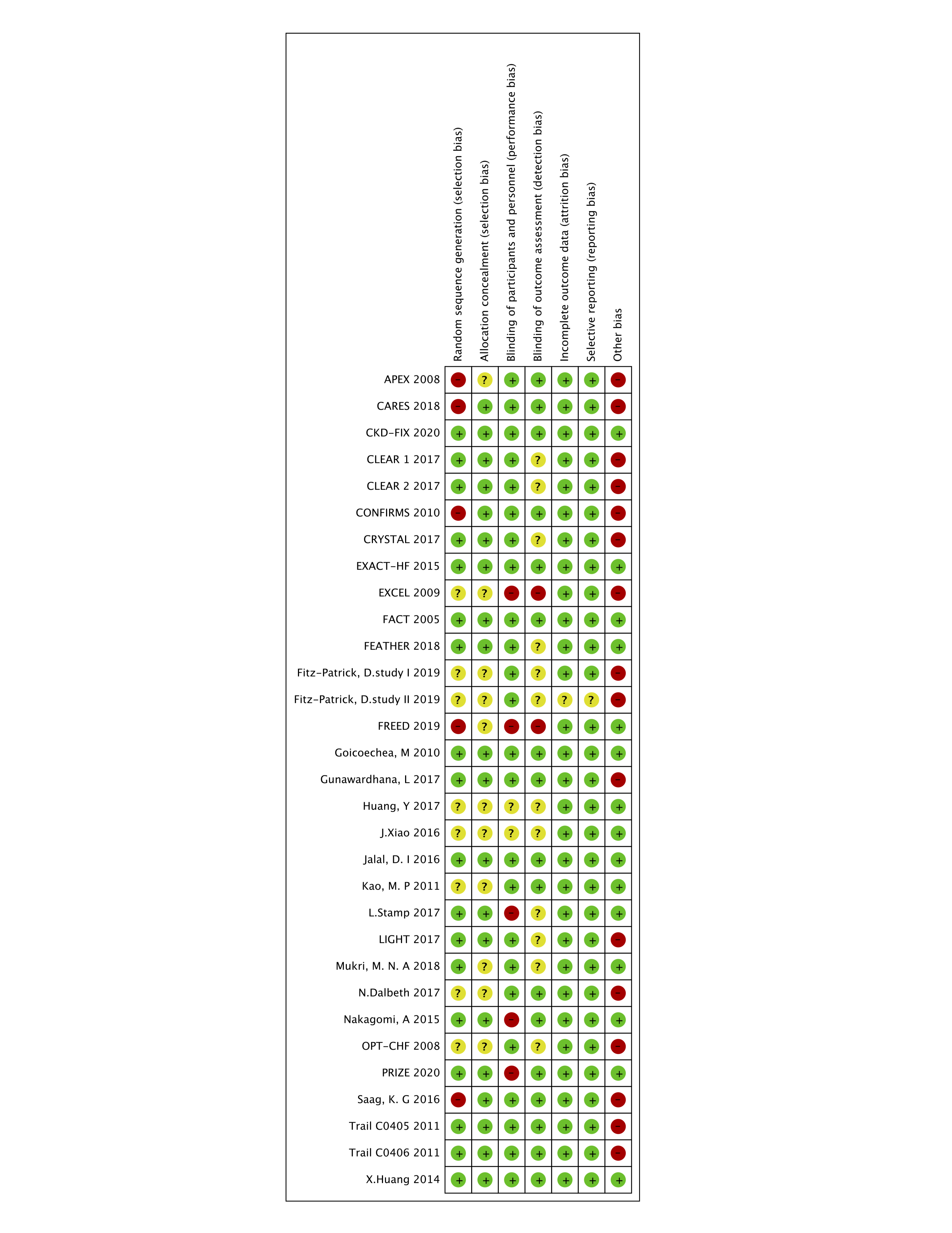

Supplement: Supplementary Figure 1 — Risk of bias graph. [file Image_1.TIF]

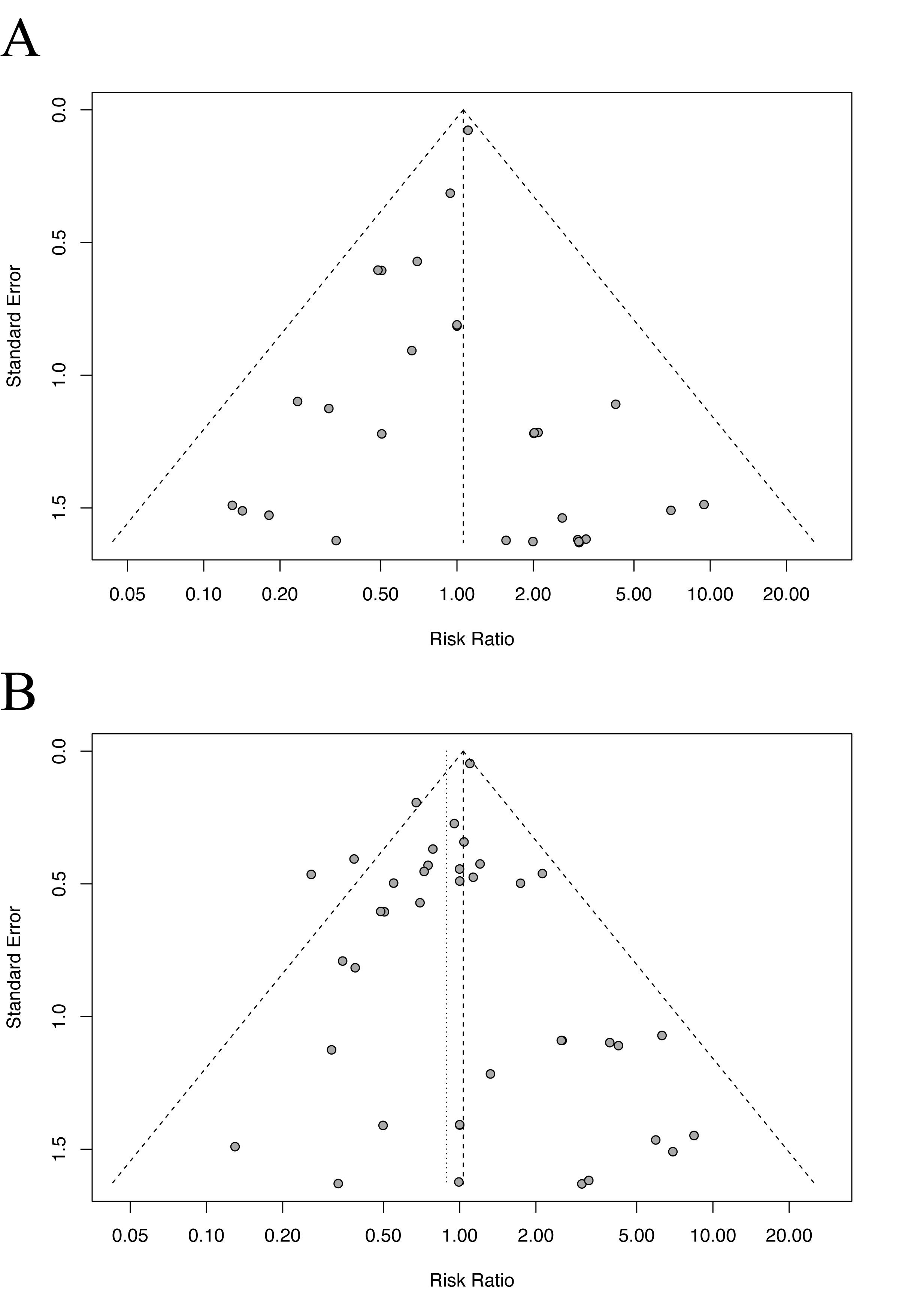

Supplement: Supplementary Figure 2 — Funnel plot of the publication bias assessment (A) MACEs (B) CVEs. [file Image_2.TIF]

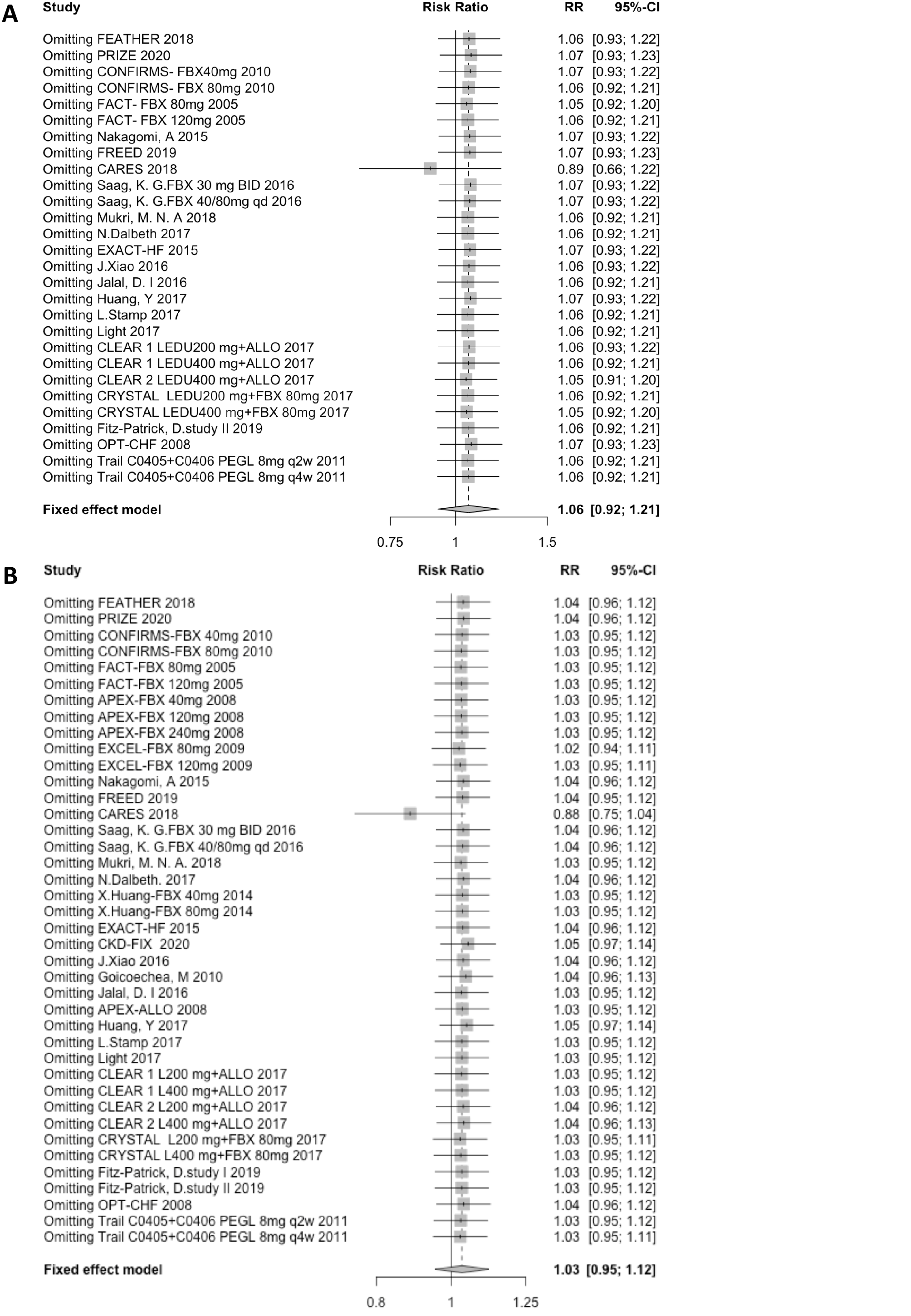

Supplement: Supplementary Figure 3 — Sensitivity analysis of the “leave-one-out” method: (A) MACEs; (B) CVEs. [file Image_3.TIF]

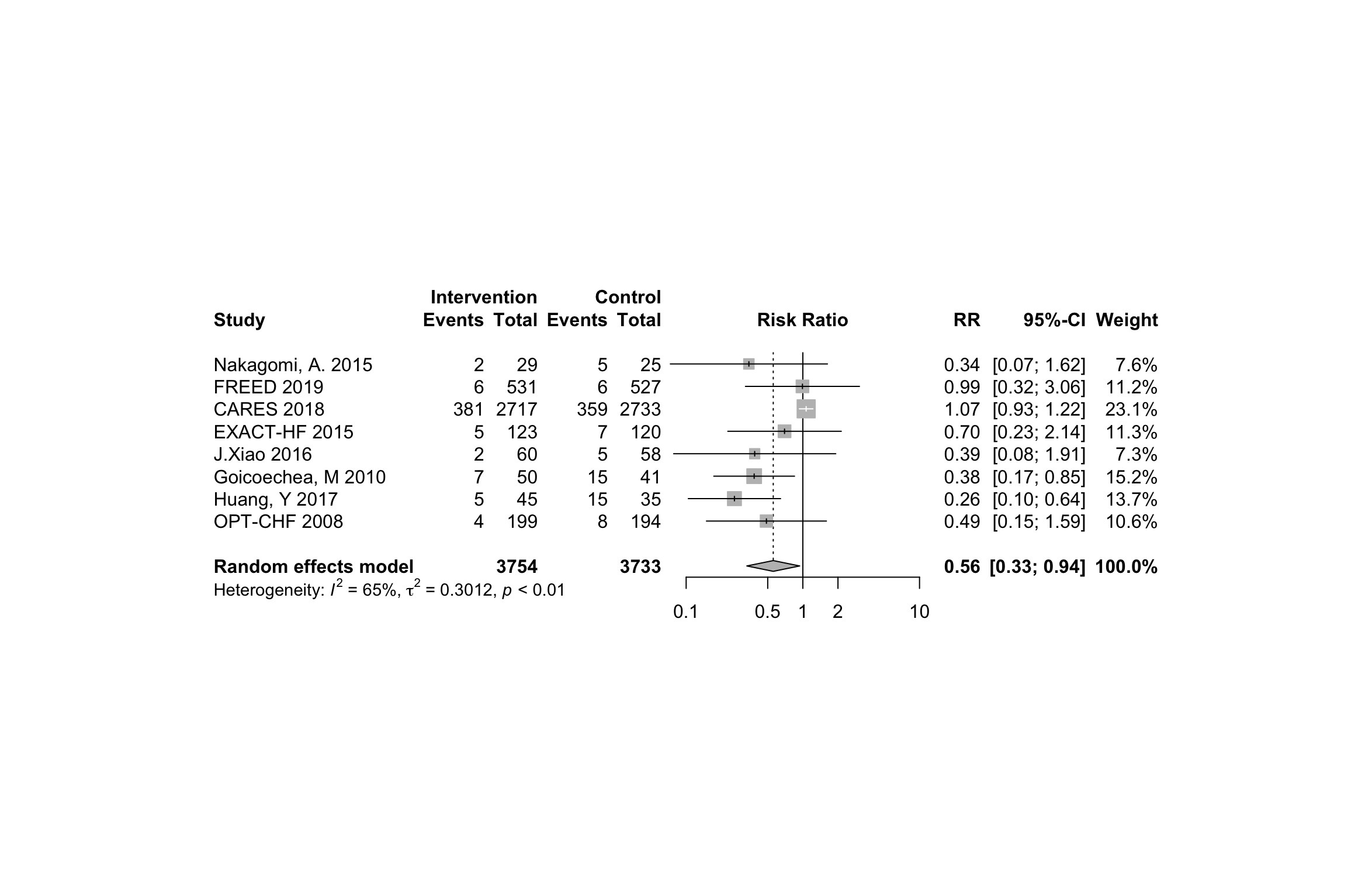

Supplement: Supplementary Figure 4 — Sensitivity analysis of the subjects with cardiovascular comorbidities. [file Image_4.TIF]

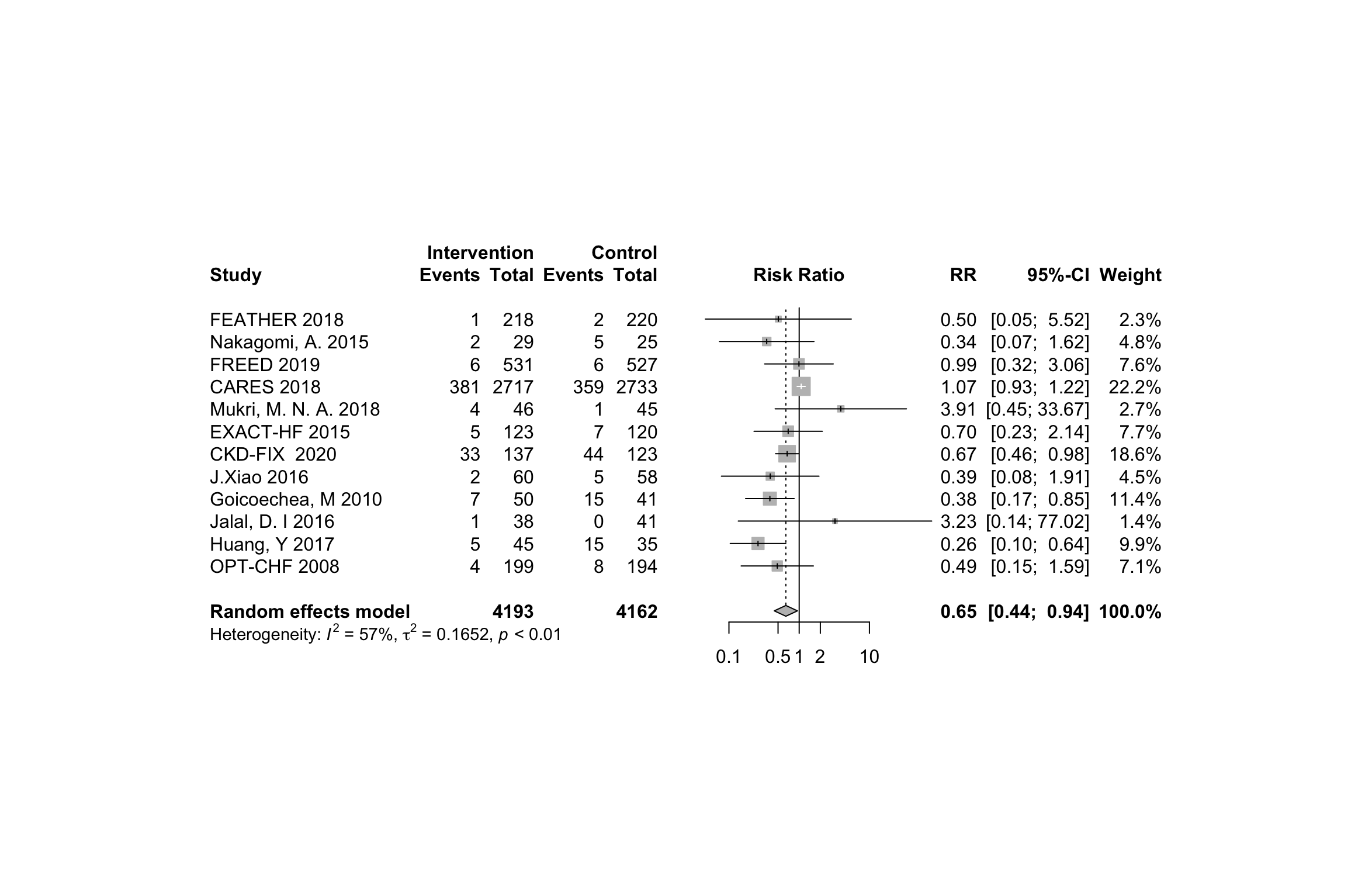

Supplement: Supplementary Figure 5 — Sensitivity analysis of the subjects with cardiorenal comorbidities. [file Image_5.TIF]

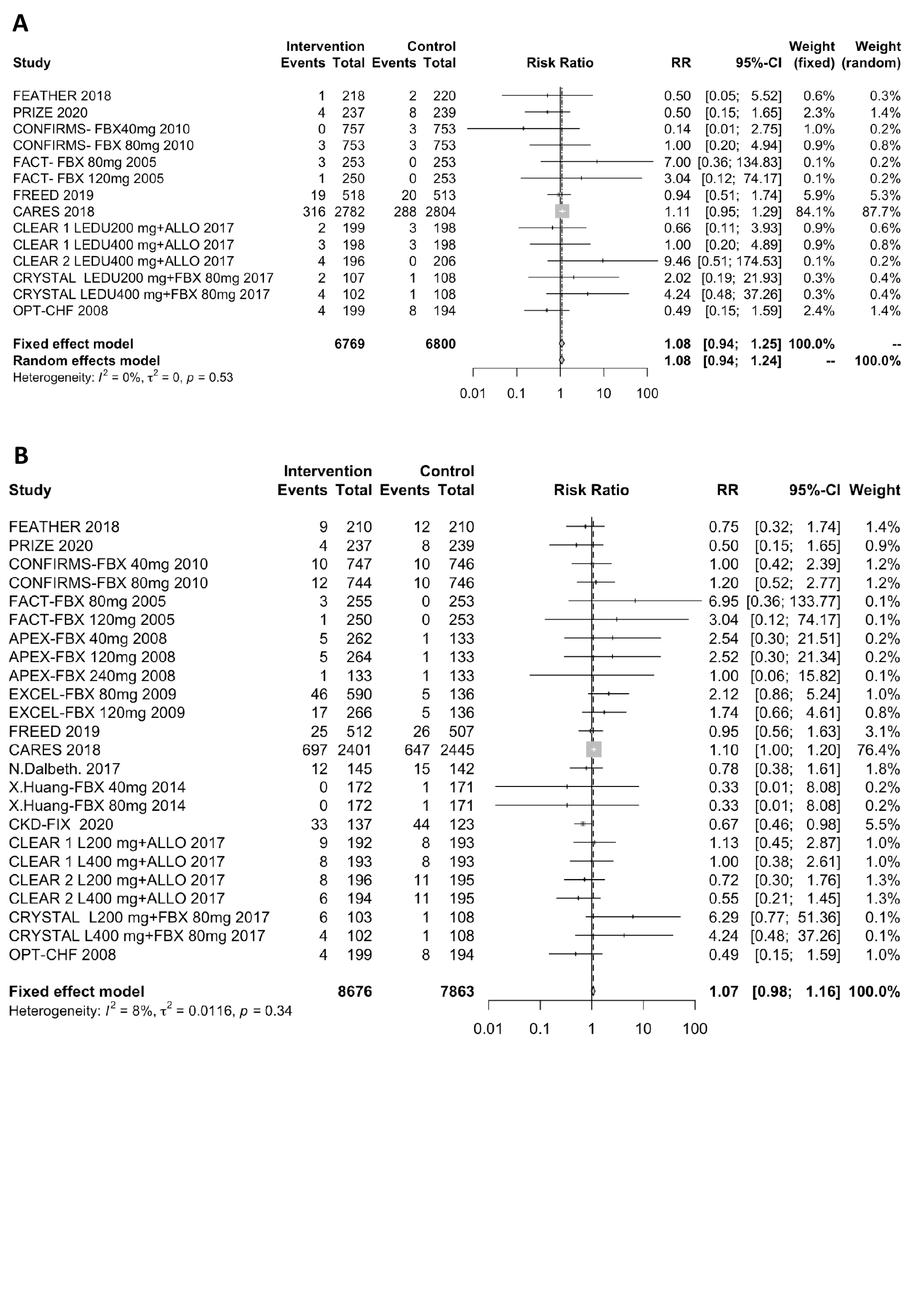

Supplement: Supplementary Figure 6 — Sensitivity analysis of the trials with a sample size ≥200 and follow-up period ≥24 w: (A) MACEs; (B) CVEs. [file Image_6.TIF]
