# Supplementary material for: Impact of Serum Uric Acid Lowering and Contemporary Uric Acid-Lowering Therapies on Cardiovascular Outcomes: A Systematic Review and Meta-Analysis
Source: Front Cardiovasc Med. 2021 Mar 23;8:641062. doi: 10.3389/fcvm.2021.641062 (PMC8044896; doi:10.3389/fcvm.2021.641062)
Supplement: Supplementary Table 1 — Baseline characteristics of the included studies. [file Table_1.DOCX]

Table S1 Baseline Characteristic of Including Studies

| Author/Trial Year | Population description | Mean Age | Males% | N | Design | Intervention Group | Control group | Major Adverse Cardiovascular Events (MACEs) | Baseline SUA (mg/dl) | Between-Group Difference in Achieved SUA (mg/dl) | Follow-up (wk) |
| --- | --- | --- | --- | --- | --- | --- | --- | --- | --- | --- | --- |
| Febuxostat | | | | | | | | | | | |
| FEATHER[1] (2018) | Hyperuricemic patients with stage 3 CKD, without Gout | 65.4 (12.0) | 77.6% | 441 | RCT | febuxostat 10-40mg qd | Placebo | Nonfatal stroke | 7.80 | 3.80 | 108 |
| PRIZE[2] (2020) | Hyperuricemia | 69.1(10.4) | 79.5% | 483 | RCT | febuxostat 10-60mg qd | Placebo | Cardiovascular death, Nonfatal myocardial infarction, Nonfatal stroke | 7.75 | 2.65 | 24 |
| CONFIRMS[3] (2010) | Hyperuricemia with gout | 52.8(11.73) | 95.4% | 2269 | RCT | febuxostat 40mg/ febuxostat 80mg | Allopurinol 200-300mg qd | Cardiovascular death, Nonfatal myocardial infarction, Nonfatal stroke | 9.57 | 0.2 (FBX 40mg)/  0.9 (FBX 80mg) | 24 |
| FACT[4] (2005) | Hyperuricemia with gout | ﻿51.8 (12.1) | 95.0% | 760 | RCT | febuxostat 80mg/ febuxostat 120mg | Allopurinol 300mg qd | Cardiovascular death | 9.84 | 1.12 (FBX 80mg) /  1.81(FBX 120mg) | 52 |
| APEX[5] (2008) | Hyperuricemia | 51.2 (11.8) | 94.0% | 1072 | RCT | febuxostat 40mg / febuxostat 120mg / febuxostat 240mg | Allopurinol 100-300mg qd /placebo | Not reported | 9.85 | 4.34 (FBX 40mg)/  5.06 (FBX 40mg)/  6.33 (FBX 120mg)/  3.04 (ALLO) | 28 |
| EXCEL[6] (2009) | Hyperuricemia | 51.2 (11.8) | 95.9% | 1086 | RCT | febuxostat 80mg/ febuxostat120mg | Allopurinol 100-300mg qd | Not reported | 9.81 | 1.43 (FBX 80mg) /  2.00 (FBX 120mg) | 173 |
| Nakagomi, A[7] (2015) | Hyperuricemic patients with CHF | 70.5 (9.1) | 71.0% | 61 | RCT | febuxostat 10-40mg qd | Allopurinol 100-300mg qd | Cardiovascular death, Nonfatal myocardial infarction | 9.35 | 0.70 | 52 |
| FREED[8] (2019) | Hyperuricemic patients with risk for any of the cerebral or CVD | 75.7 (6.6) | 69.1% | 1070 | RCT | febuxostat 10-40mg qd | non-febuxostat | Cardiovascular death, Nonfatal myocardial infarction, Nonfatal stroke | 7.52 | 2.30 | 156 |
| CARES[9] (2018) | Hyperuricemia with gout, history of cardiovascular or cerebro-vascular disease | 64.8 (8.53) | 84.1% | 6190 | RCT | febuxostat 40-80mg qd | Allopurinol 300-600mg qd | Cardiovascular death, Nonfatal myocardial infarction, Nonfatal stroke | 8.70 | NA | 365 |
| Gunawardhana, L[10] (2017) | Hyperuricemic patients with Hypertension | 53.6 (10.6) | 82.0% | 121 | RCT | febuxostat 80mg qd | Placebo | Not reported | 7.65 | 3.40 | 6 |
| Saag, K. G[11] (2016) | hyperuricemia | 65.7 (10.6) | 78.1% | 96 | RCT | Febuxostat 30 mg BID/febuxostat 40/80mg qd | Placebo | Cardiovascular death | 10.5 | 4.82 (FBX 60mg)/  4.02 (FBX40/80mg) | 52 |
| Mukri, M. N. A[12] (2018) | Hyperuricemia with CKD stages 3 and 4 | 65.5 (8.4) | 53.0% | 93 | RCT | febuxostat 40mg qd | Placebo | Cardiovascular death | 9.11 | 3.51 | 24 |
| N. Dalbeth[13] (2017) | Hyperuricemia with gout | 50.8 (12.1) | 91.1% | 314 | RCT | febuxostat 40mg or 80mg qd | Placebo | Cardiovascular death, Nonfatal myocardial infarction, Nonfatal stroke | 8.75 | 2.40 | 104 |
| X.Huang[14] (2014) | Hyperuricemia with gout | 46.7 (11.4) | 97.1% | 516 | RCT | febuxostat 40mg/ febuxostat80mg | Allopurinol 300mg qd | Not reported | 9.94 | 0 (FBX 40mg)/  0.92 (FBX 80mg) | 28 |
| Allopurinol | | | | | | | | | | | |
| EXACT-HF[15] (2015) | Hyperuricemic Heart Failure Patients | 61.9(13.6) | 86.0% | 253 | RCT | Allopurinol 300-600mg qd | Placebo | Cardiovascular death | 11.1 | 4.30 | 24 |
| CKD-FIX[16] (2020) | CKD stages 3 and 4 without gout | 62.1 (12.7) | 62.0% | 363 | RCT | Allopurinol 100-300mg qd | Placebo | Not reported | 8.20 | 2.90 | 104 |
| J. Xiao[17] (2016) | Non-hyperuricemic patients with CHF | 52.1 (14.0) | 72.6% | 125 | RCT | Allopurinol 300mg qd | Placebo | Cardiovascular death | 5.40 | 0.37 | 24 |
| Goicoechea, M[18] (2010) | CKD, eGFR＜60 | 71.7 (8.7) | NA | 113 | RCT | Allopurinol 100mg qd | Placebo | Not reported | 7.55 | 2.00 | 104 |
| Kao, M. P[19] (2011) | CKD stages 3 with LVH | 72.1(6.3) | 59.0% | 53 | RCT | ﻿Allopurinol 300mg qd | Placebo | Not reported | 7.23 | 3.37 | 39 |
| Jalal, D. I[20] (2016) | CKD stages 3 with hyperuricemia | 57.4(11.7) | 82.0% | 80 | RCT | Allopurinol 300mg qd | Placebo | Cardiovascular death | 8.50 | 3.29 | 12 |
| Huang, Y[21] (2017) | ACS | 56.3(5.2) | 60.0% | 100 | RCT | Allopurinol 600mg-200mg qd | Placebo | Cardiovascular death, Nonfatal myocardial infarction | 9.82 | 0.99 | 104 |
| L.Stamp[22] (2017) | hyperuricemia with gout | 60.2(12.4) | 91.0% | 183 | RCT | Allopurinol dose escalation | Placebo | Cardiovascular death | 7.15 | 1.16 | 52 |
| Lesinurad | | | | | | | | | | | |
| LIGHT[23] (2017) | hyperuricemia with gout | 54.4(12.2) | 91.6% | 214 | RCT | lesinurad 400mg qd | Placebo | Cardiovascular death | 9.33 | NA | 24 |
| CLEAR 1[24] (2017) | hyperuricemia with gout | 51.9(11.3) | 95.5% | 603 | RCT | lesinurad 200 mg + allopurinol/ lesinurad 400 mg + allopurinol | Placebo + Allopurinol | Cardiovascular death, Nonfatal myocardial infarction, Nonfatal stroke | 6.94 | NA | 52 |
| CLEAR 2[25] (2017) | hyperuricemia with gout | 51.2(10.9) | 96.6% | 610 | RCT | lesinurad 200 mg + allopurinol/  lesinurad 400 mg + allopurinol | Placebo + Allopurinol | Cardiovascular death, Nonfatal myocardial infarction, Nonfatal stroke | 6.90 | NA | 52 |
| CRYSTAL[26] (2017) | hyperuricemia with gout | 54.1(11.0) | 94.3% | 324 | RCT | Lesinurad 200 mg + Febuxostat 80 mg/ Lesinurad 400 mg + Febuxostat 80 mg | Placebo + Febuxostat | Cardiovascular death, Nonfatal myocardial infarction, Nonfatal stroke | 8.70 | NA | 52 |
| Verinurad | | | | | | | | | | | |
| Fitz-Patrick, D. study I[27] (2019) | Hyperuricemia with gout | 53.7(9.5) | 93.0% | 171 | RCT | verinurad 5-12.5mg qd | Placebo | Not reported | 8.56 | 2.29 | 24 |
| Fitz-Patrick, D. study II[27] (2019) | Hyperuricemia | 52.3(10.02) | 100.0% | 204 | RCT | verinurad 2.5-15mg qd | Allopurinol/ Placebo | Nonfatal myocardial infarction | 8.52 | 2.01 | 24 |
| Oxypurinol | | | | | | | | | | | |
| OPT-CHF[28] (2008) | heart failure (Stable NYHA Class III-IV) | 64.5(13) | 76.0% | 405 | RCT | Oxypurinol 600mg qd | Placebo | Cardiovascular death | 7.90 | NA | 24 |
| Pegloticase | | | | | | | | | | | |
| Trail C0405+ Trail C0406[29] (2011) | Hyperuricemia with gout | 55.4(14.01) | 81.6% | 212 | RCT | pegloticase 8mg q2w/ pegloticase 8mg q4w | Placebo | Cardiovascular death, Nonfatal myocardial infarction | 9.78 | NA | 24 |

Table S1 Baseline Characteristic of Including Studies

Abbreviations:

MACEs, Major Adverse Cardiovascular Events

CKD, chronic kidney disease

FBX, febuxostat

ALLO, allopurinol

CHF, chronic heart failure

CVD, cardiovascular disease

eGFR, estimated glomerular filtration rate

ACS, acute coronary syndrome

LVH, left ventricular hypertrophy

[1] K. Kimura *et al.*, “Febuxostat Therapy for Patients With Stage 3 CKD and Asymptomatic Hyperuricemia: A Randomized Trial,” *Am. J. Kidney Dis.*, vol. 72, no. 6, pp. 798–810, 2018, doi: 10.1053/j.ajkd.2018.06.028.

[2] A. Tanaka *et al.*, “Febuxostat does not delay progression of carotid atherosclerosis in patients with asymptomatic hyperuricemia: A randomized, controlled trial,” *PLoS Med.*, vol. 17, no. 4, p. e1003095, 2020, doi: 10.1371/journal.pmed.1003095.

[3] M. A. Becker *et al.*, “The urate-lowering efficacy and safety of febuxostat in the treatment of the hyperuricemia of gout: The CONFIRMS trial,” *Arthritis Res. Ther.*, vol. 12, no. 2, pp. 16–18, 2010, doi: 10.1186/ar2978.

[4] M. A. Becker *et al.*, “Febuxostat compared with allopurinol in patients with hyperuricemia and gout,” *N. Engl. J. Med.*, vol. 353, no. 23, pp. 2450–2461, 2005, doi: 10.1056/NEJMoa050373.

[5] H. R. Schumacher *et al.*, “Effects of febuxostat versus allopurinol and placebo in reducing serum urate in subjects with hyperuricemia and gout: A 28-week, phase III, randomized, double-blind, parallel-group trial,” *Arthritis Care Res.*, vol. 59, no. 11, pp. 1540–1548, 2008, doi: 10.1002/art.24209.

[6] M. A. Becker, H. R. Schumacher, P. A. MacDonald, E. Lloyd, and C. Lademacher, “Clinical efficacy and safety of successful longterm urate lowering with febuxostat or allopurinol in subjects with gout,” *J. Rheumatol.*, vol. 36, no. 6, pp. 1273–1282, 2009, doi: 10.3899/jrheum.080814.

[7] A. Nakagomi *et al.*, “Effects of febuxostat and allopurinol on the inflammation and cardiac function in chronic heart failure patients with hyperuricemia,” *IJC Metab. Endocr.*, vol. 8, pp. 46–55, 2015, doi: 10.1016/j.ijcme.2015.07.001.

[8] S. Kojima *et al.*, “Febuxostat for cerebral and cardiorenovascular events prevention study,” *Eur. Heart J.*, vol. 40, no. 22, pp. 1778-1786A, 2019, doi: 10.1093/eurheartj/ehz119.

[9] W. B. White *et al.*, “Cardiovascular safety of febuxostat or allopurinol in patients with gout,” *N. Engl. J. Med.*, vol. 378, no. 13, pp. 1200–1210, 2018, doi: 10.1056/NEJMoa1710895.

[10] L. Gunawardhana *et al.*, “Effect of Febuxostat on ambulatory blood pressure in subjects with hyperuricemia and hypertension: A Phase 2 randomized placebo-controlled study,” *J. Am. Heart Assoc.*, vol. 6, no. 11, 2017, doi: 10.1161/JAHA.117.006683.

[11] K. G. Saag, A. Whelton, M. A. Becker, P. MacDonald, B. Hunt, and L. Gunawardhana, “Impact of Febuxostat on Renal Function in Gout Patients With Moderate-to-Severe Renal Impairment,” *Arthritis Rheumatol.*, vol. 68, no. 8, pp. 2035–2043, 2016, doi: 10.1002/art.39654.

[12] M. N. A. Mukri *et al.*, “Role of febuxostat in retarding progression of diabetic kidney disease with asymptomatic hyperuricemia: A 6-months open-label, randomized controlled trial,” *EXCLI J.*, vol. 17, pp. 563–575, 2018, doi: 10.17179/excli2018-1256.

[13] N. Dalbeth *et al.*, “Effects of Febuxostat in Early Gout: A Randomized, Double-Blind, Placebo-Controlled Study,” *Arthritis Rheumatol.*, vol. 69, no. 12, pp. 2386–2395, 2017, doi: 10.1002/art.40233.

[14] X. Huang *et al.*, “An allopurinol-controlled, multicenter, randomized, double-blind, parallel between-group, comparative study of febuxostat in Chinese patients with gout and hyperuricemia,” *Int. J. Rheum. Dis.*, vol. 17, no. 6, pp. 679–686, 2014, doi: 10.1111/1756-185X.12266.

[15] M. M. Givertz *et al.*, “Effects of xanthine oxidase inhibition in hyperuricemic heart failure patients: The xanthine oxidase inhibition for hyperuricemic heart failure patients (EXACT-HF) study,” *Circulation*, vol. 131, no. 20, pp. 1763–1771, 2015, doi: 10.1161/CIRCULATIONAHA.114.014536.

[16] S. V. Badve *et al.*, “Effects of Allopurinol on the Progression of Chronic Kidney Disease,” *N. Engl. J. Med.*, vol. 382, no. 26, pp. 2504–2513, 2020, doi: 10.1056/NEJMoa1915833.

[17] J. Xiao *et al.*, “Allopurinol ameliorates cardiac function in non-hyperuricaemic patients with chronic heart failure,” *Eur. Rev. Med. Pharmacol. Sci.*, vol. 20, no. 4, pp. 756–761, 2016.

[18] M. Goicoechea *et al.*, “Effect of allopurinol in chronic kidney disease progression and cardiovascular risk,” *Clin. J. Am. Soc. Nephrol.*, vol. 5, no. 8, pp. 1388–1393, 2010, doi: 10.2215/CJN.01580210.

[19] M. P. Kao *et al.*, “Allopurinol benefits left ventricular mass and endothelial dysfunction in chronic kidney disease,” *J. Am. Soc. Nephrol.*, vol. 22, no. 7, pp. 1382–1389, 2011, doi: 10.1681/ASN.2010111185.

[20] D. I. Jalal *et al.*, “Vascular function and uric acid-lowering in stage 3 CKD,” *J. Am. Soc. Nephrol.*, vol. 28, no. 3, pp. 943–952, 2017, doi: 10.1681/ASN.2016050521.

[21] Y. Huang *et al.*, “Clinical Study on efficacy of allopurinol in patients with acute coronary syndrome and its functional mechanism,” *Hell. J. Cardiol.*, vol. 58, no. 5, pp. 360–365, 2017, doi: 10.1016/j.hjc.2017.01.004.

[22] L. K. Stamp *et al.*, “A randomised controlled trial of the efficacy and safety of allopurinol dose escalation to achieve target serum urate in people with gout,” *Ann. Rheum. Dis.*, vol. 76, no. 9, pp. 1522–1528, 2017, doi: 10.1136/annrheumdis-2016-210872.

[23] A. K. Tausche *et al.*, “Lesinurad monotherapy in gout patients intolerant to a xanthine oxidase inhibitor: A 6 month phase 3 clinical trial and extension study,” *Rheumatol. (United Kingdom)*, vol. 56, no. 12, pp. 2170–2178, 2017, doi: 10.1093/rheumatology/kex350.

[24] K. G. Saag *et al.*, “Lesinurad Combined With Allopurinol: A Randomized, Double-Blind, Placebo-Controlled Study in Gout Patients With an Inadequate Response to Standard-of-Care Allopurinol (a US-Based Study),” *Arthritis Rheumatol.*, vol. 69, no. 1, pp. 203–212, 2017, doi: 10.1002/art.39840.

[25] T. Bardin *et al.*, “Lesinurad in combination with allopurinol: A randomised, double-blind, placebo-controlled study in patients with gout with inadequate response to standard of care (the multinational CLEAR 2 study),” *Ann. Rheum. Dis.*, vol. 76, no. 5, pp. 811–820, 2017, doi: 10.1136/annrheumdis-2016-209213.

[26] N. Dalbeth *et al.*, “Lesinurad, a Selective Uric Acid Reabsorption Inhibitor, in Combination With Febuxostat in Patients With Tophaceous Gout: Findings of a Phase III Clinical Trial,” *Arthritis Rheumatol.*, vol. 69, no. 9, pp. 1903–1913, 2017, doi: 10.1002/art.40159.

[27] D. Fitz-Patrick *et al.*, “Safety and efficacy of verinurad, a selective URAT1 inhibitor, for the treatment of patients with gout and/or asymptomatic hyperuricemia in the United States and Japan: Findings from two phase II trials,” *Mod. Rheumatol.*, vol. 29, no. 6, pp. 1042–1052, 2019, doi: 10.1080/14397595.2018.1538003.

[28] J. M. Hare *et al.*, “Impact of Oxypurinol in Patients With Symptomatic Heart Failure. Results of the OPT-CHF Study,” *J. Am. Coll. Cardiol.*, vol. 51, no. 24, pp. 2301–2309, 2008, doi: 10.1016/j.jacc.2008.01.068.

[29] J. S. Sundy *et al.*, “Efficacy and tolerability of pegloticase for the treatment of chronic gout in patients refractory to conventional treatment: Two randomized controlled trials,” *JAMA - J. Am. Med. Assoc.*, vol. 306, no. 7, pp. 711–720, 2011, doi: 10.1001/jama.2011.1169.
