# Supplementary material for: Impact of Serum Uric Acid Lowering and Contemporary Uric Acid-Lowering Therapies on Cardiovascular Outcomes: A Systematic Review and Meta-Analysis
Source: Front Cardiovasc Med. 2021 Mar 23;8:641062. doi: 10.3389/fcvm.2021.641062 (PMC8044896; doi:10.3389/fcvm.2021.641062)
Supplement: Supplementary Table 2 — Search strategy. [file Table_2.DOCX]

Table S2

Search strategy

1. Hyperuricemia [tiab]

2. "uric acid" [tiab]

3. ("uric acid" or urate (elevat* or high or raise* or rise or rising)) [tiab]

4. #1 OR #2 OR #3

5.uric acid-lowering [tiab]

6. UA-lowering [tiab]

7. lowering [tiab]

8. #5 OR #6 OR (#7 AND #2)

9. (xanthine oxidase inhibit*) [tiab]

10.allopurinol [tiab]

11. febuxostat [tiab]

12. oxypurinol [tiab]

13. topiroxosta [tiab]

14. RLBN1001 [tiab]

15. KUX-1151 [tiab]

16. #9 OR #10 OR #11 OR #12 OR #13 OR #14 OR #15

17. (uricosuric agent*) [tiab]

18. Benzbromarone [tiab]

19. probenecid [tiab]

20. Sulfinpyrazone [tiab]

21. Lesinurad [tiab]

22.RDEA594 [tiab]

23. Verinurad [tiab]

24. RDEA3170 [tiab]

25. Arhalofenate [tiab]

26.MBX-102 [tiab]

27. Tranilast [tiab]

28. Levotofisopam [tiab]

29. UR-1102 [tiab]

30. #17 OR #18 OR #19 OR #20 OR #21 OR #22 OR #23 OR #24 OR #25 OR #26 OR #27 OR #28 OR #29

31. uricase [tiab]

32.Pegloticase [tiab]

33. Krystexxa [tiab]

34. rasburicase [tiab]

35. #31 OR #32 OR #33 OR #34

36. Ulodesine [tiab]

37. BCX4208 [tiab]

38.(Purine nucleotide phosphorylasc inhibitor*) [tiab]

39. #36 OR #37 OR #38

40.Duzallo [tiab]

41.apazone [tiab]

42.Zoxazolamine [tiab]

43. #40 OR #41 OR #42

44.major adverse cardiovascular event [All Fields]

45.MACE [All Fields]

46.atherosclerosis [All Fields]

47.death [All Fields]

48.mortality [All Fields]

49.stroke [All Fields]

50.heart failure [All Fields]

51.coronary [All Fields]

52.cardiovascular [All Fields]

53.﻿cardiac [All Fields]

54.Cerebrovascular Disorders [All Fields]

55.Cardiovascular Diseases [All Fields]

56.angina [All Fields]

57.﻿revascularization [All Fields]

58. #44 OR #45 OR #46 OR #47 OR #48 OR #49 OR #50 OR #51 OR #52 OR #53 OR #54 OR #55 OR #56 OR #57

58.randomized controlled trial [pt]

59.controlled clinical trial [pt]

60.randomized [tiab]

61.placebo[tiab]

62.drug therapy [sh]

63.randomly [tiab]

64.trial [tiab]

65.groups [tiab]

66. #58 OR #59 OR #60 OR #61 OR #62 OR #63 OR #64 OR #65

67. animals [mh] NOT humans [mh]

68. #66 NOT #67

69. #4 AND (#8 OR#16 OR #30 OR #35 OR #39 OR #43) AND #58 AND #68
